# Disseminated *Mycobacterium intracellulare* subsp. *chimaera* infection, undiagnosed for years, highlights the enduring clinical utility of “old school” microbiological testing and a robust differential

**DOI:** 10.1128/asmcr.00091-25

**Published:** 2025-12-12

**Authors:** Ayesha Khan

**Affiliations:** 1 Division of Clinical Microbiology, Department of Pathology and Laboratory Medicine, UC Irvine School of Medicine, Orange, California, USA; Pattern Bioscience, Austin, Texas, USA

## Abstract

Disseminated *Mycobacterium intracellulare* subsp. *chimaera* (MC) infections are rare, slow-progressing, and easily overlooked, particularly when a patient’s history of prior cardiac surgery is not incorporated into the diagnostic evaluation. In a recent *ASM Case Reports* article (1:e00003-25, 2025, https://doi.org/10.1128/asmcr.00003-25), Ladines-Lim et al. describe a disseminated MC infection in a patient with prior aortic and mitral valve replacement that remained undiagnosed for over 4 years. Conventional microbiological testing was not pursued early in the course of illness because the history of cardiopulmonary bypass was not linked with the constellation of unexplained symptoms. This case urges clinicians to remain vigilant and suspect MC in patients with prior open-chest cardiac surgery who present with gradually worsening, systemic symptoms. Since 2013, global outbreaks of delayed-onset MC infections have been traced to contaminated heater cooler devices, yet many centers continue to face barriers to replacing or monitoring such equipment. A delayed diagnosis in this case was eventually established by cell-free metagenomic next-generation sequencing (cfmNGS). However, the result was not acted upon until weeks later, after central nervous system involvement. A more timely, cost-effective diagnosis might have been achieved using traditional, widely available, culture-based testing guided by a robust exposure-driven differential. Clinicians should suspect MC in patients with prior cardiac surgery—even years earlier—who develop unexplained, progressive systemic symptoms. Early suspicion and appropriate testing are critical to improved outcomes. This case shows that next-generation sequencing assays are only as useful as the clinical reasoning guiding their use. Traditional microbiological testing—when leveraged early and thoughtfully—remains an accessible cornerstone of diagnosing complex MC infections.

## COMMENTARY

In a paper published in *ASM Case Reports*, Ladines-Lim et al. (1) describe a striking case of disseminated *Mycobacterium intracellulare* subsp. *chimaera* (MC) infection in a patient with prior open-chest cardiac surgery. The infection was undiagnosed for over 4 years, despite progressive multisystem disease, because MC was not included in the differential diagnosis, and conventional microbiological testing was not performed. Only after repeated hospitalizations and worsening disease—including central nervous system (CNS) involvement—was the diagnosis confirmed with cell-free metagenomic next-generation sequencing (cfmNGS). This delayed diagnosis underscores the importance of linking unexplained systemic symptoms with prior cardiopulmonary bypass (CPB) exposure, particularly in light of well-documented global outbreaks of delayed-onset, invasive MC infections associated with contaminated heater-cooler devices (HCDs) (2–4). Many hospitals and centers in low-resource settings encounter severe systemic inequities and barriers to replace and monitor such equipment (5).

**Fig 1:**
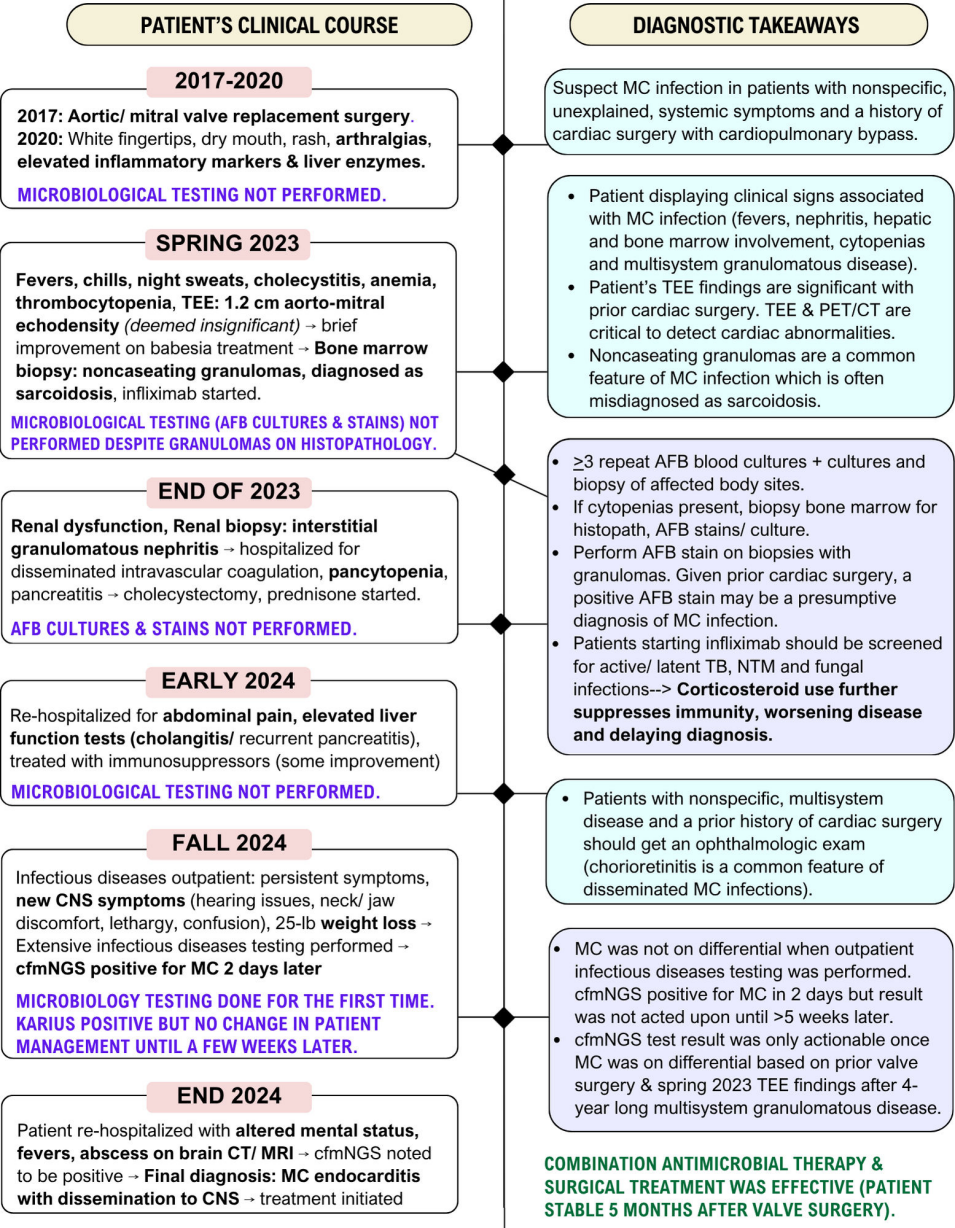
Overview of the patient case described by Ladines-Lim et al. with clinical course of illness summarized on the left, and key diagnostic takeaways on the right. Bolded words (black font) are clinical features and findings associated with *Mycobacterium intracellulare* subsp. *chimaera* (MC) infections (2, 3, 6, 7). Blue boxes indicate high-yield diagnostic tools based on telltale clinical signs that can facilitate a timely diagnosis. Purple boxes indicate recommended microbiological testing.

The patient’s clinical course (2020–2024) highlights key diagnostic pitfalls (Fig. 1). Noncaseating granulomas were identified on biopsy, but acid-fast staining and culture were not performed, leading to a misdiagnosis of sarcoidosis and inappropriate immunosuppressive therapy. A transesophageal echocardiogram later revealed aortic-mitral abnormalities suggestive of endocarditis, but these findings were initially dismissed. When the outpatient cfmNGS test returned positive for MC, there was no immediate follow-up. The result was only acted upon weeks later, after the patient was rehospitalized with worsening CNS involvement.

This sequence of events shows that even advanced diagnostics have limited utility and impact on patients when not anchored to a robust clinical differential, and that delays in follow-up can render otherwise actionable results ineffectual. Importantly, if “old school” microbiological testing, such as mycobacterial (acid fast bacilli; AFB) cultures and stains, had been ordered earlier—guided by a exposure-driven differential—a diagnosis likely could have been established sooner, prompting earlier initiation of therapy. These conventional testing tools are cost-effective, are widely accessible, and can provide clinically actionable results when deployed appropriately (6, 7) (Fig. 1 and Table 1).

**TABLE 1:** Common clinical presentations, symptoms, and findings of *Mycobacterium intracellulare* subsp. *chimaera* (MC) infection with high yield testing and antimicrobial therapy recommendations from evidence-based guidelines (2, 3, 8, 9)

Category	
Exposure	History of open-chest cardiac surgery with cardiopulmonary bypass (prosthetic implantations carry the highest risk)
Time from surgery to onset of symptoms	Reported average 1.5–3 years, up to 6–7 years
Cardiac manifestations	Endocarditis related to prostheses (prosthetic heart valve, prosthetic graft infection, mechanical circulatory support device) Myocarditis Pseudoaneurysm formation Localized sternal/thoracic wound infection (less common) Mediastinitis
Extracardiac or extrathoracic manifestations	Bloodstream infection/disseminated infection (common) Bone marrow involvement Splenomegaly Bone infection (arthritis, osteomyelitis, spinal involvement) Pneumonitis Hepatitis Nephritis Chorioretinitis (common sign of disseminated infection) Cerebral vasculitis
Clinical symptoms	Recurrent, prolonged fevers of unknown origin Malaise, lethargy Weight loss Night sweats, chills Arthralgias/myalgias Cough, dyspnea (less common) Chest pain (less common)
Physical findings	Frequently normal Heart murmur Hepatosplenomegaly Signs of localized sternal surgical site infection Chorioretinitis (common in disseminated infections)
Laboratory findings	Lymphopenia Thrombocytopenia Anemia Elevated liver enzymes Elevated inflammatory markers
Histopathological findings	Granulomatous infection with multiorgan involvement (noncaseating granulomas and foamy, enlarged macrophages are common on biopsies but not specific to MC and can be seen in *Mycobacterium tuberculosis* or other fungal infections) Variable AFB smear results
Common misdiagnosis	Sarcoidosis
Recommended imaging	Transesophageal echocardiography (more sensitive than transthoracic echocardiography) Positron emission tomography and computed tomography often needed to detect prosthetic valve or aortic graft involvement and determine extent of dissemination
Initial microbiological testing is recommended	≥3 repeat AFB blood cultures AFB culture and histopathology on specimens from other involved body sites (tissue/fluid/abscess, explanted prosthetic material, purulent material in sternal/localized infections) AFB stains on specimens sent for culture and histopathology
Follow-up or second-line microbiological testing if it is accessible (guided by a clinical hypothesis and robust differential)	If cultures grow an organism identified as *Mycobacterium avium-intracellulare* complex, sequencing is required to confirm identification of MC species (*16-23S ITS, rpoB, hsp65*) Direct from sample, targeted, mycobacterial sequencing can be performed on fresh tissue/fluid (preferred) or formalin-fixed paraffin-embedded tissue (highest yield when AFB and/or noncaseating granulomas are seen on specimen) Metagenomic sequencing-based test (Karius)
Other high-yield testing is recommended	Bone marrow biopsy if cytopenias are present Ophthalmologic examination (chorioretinal lesions are a common finding in disseminated MC infection)
Antimicrobial susceptibility testing Note: therapeutic drug monitoring and monitoring for adverse reactions are necessary (e.g., renal function, vestibular function, audiograms, ophthalmologic exams)	General recommendations: Should be performed at experienced and/or reference laboratories If not performed at baseline, isolates should be saved in the event of a recurrent/refractory infection Recommended MIC testing: Clarithromycin (predicts susceptibility to azithromycin, correlates relatively well with treatment outcomes) Amikacin (to optimize dosing)
Antimicrobial therapy (often used in conjunction with surgical treatment) Note: Corticosteroid therapy and TNF-alpha inhibitors further suppress immunity, worsen disease progression, and delay diagnosis.	Combination therapy with 3–4 agents: Macrolide, rifamycin, ethambutol, and amikacin (if tolerated) Second-line agents (if isolate is resistant to macrolides or amikacin, toxicity to first-line agents, or refractory infections): Preferred: Clofazimine (FDA-approved but not commercially available in the United States, requires compassionate use approval and access to drug from the manufacturer) Options with limited clinical data and/or risk of elevated MICs *in vitro*: Linezolid, moxifloxacin, bedaquiline

Figure 1 summarizes the patient’s clinical course alongside diagnostic takeaways, while Table 1 outlines typical clinical features of MC infection, high-yield testing, and treatment considerations (2, 3, 8). In addition to case series and outbreak investigations, the 2020 International Society of Cardiovascular Infectious Diseases guidelines are a valuable resource (2, 3, 6, 7, 10–15).

## CLINICAL TAKEAWAYS

Patients with MC infection often present with nonspecific, slowly progressive symptoms, such as fevers, cytopenias, hepatic or renal involvement, and arthralgias (6, 7) (Table 1). Ophthalmologic exam and cardiac imaging may provide early clues. A transesophageal echocardiogram and nuclear imaging with positron emission tomography and computed tomography scans are often required to detect prosthetic valve involvement or graft infections. However, extrathoracic symptoms can precede cardiac abnormalities, and normal imaging cannot rule out MC infection. MC infections are often misdiagnosed as sarcoidosis due to multisystem granulomatous inflammation, contributing to delayed diagnosis (16–18). These subtleties reinforce the need for vigilance in patients with prior CPB exposure, including those presenting with suspected autoimmune disorders. Management is complex and often requires prolonged multidrug therapy with surgical intervention (3, 6). Culture-based methods have the advantage of enabling phenotypic antimicrobial susceptibility testing (19). Clarithromycin susceptibility testing, which can also predict susceptibility to azithromycin, has been shown to correlate with treatment outcomes (19–22).

## MICROBIOLOGY TESTING TAKEAWAYS

Timely microbiological testing is essential for optimal diagnosis and management of MC infections. AFB blood cultures have ~70%–90% sensitivity, with enhanced yield when ≥3 sets are obtained (2, 3, 12, 15, 17). Sampling of multiple involved body sites increases sensitivity. Tissue biopsies should be sent for histopathology, AFB culture, and AFB stains. Negative stain does not rule out MC. Noncaseating granulomas remain a common histopathological feature of MC infections (11, 17, 23, 24). Targeted broad-range mycobacterial sequencing may be useful on fresh tissue and when organisms are visualized in histopathology samples, but is costly and not widely available.

Definitive mycobacterial species identification requires sequencing (16S-23S ITS, *rpoB, hsp65*) (25). However, in practice, isolation of MAC from blood, tissue, or prosthetic material in a patient with prior CPB exposure is often sufficient to initiate treatment. While culture turnaround times are long, early suspicion enables timely testing, balancing delays with the accessibility and affordability of traditional microbiological methods.

## NEXT-GENERATION SEQUENCING ASSAYS

This case also offers broader lessons on the role of NGS in clinical practice. Although rapidly advancing NGS technologies hold great promise, their clinical utility is highly contextual. NGS assays should be reserved for select scenarios: diagnosis of elusive infections in high-risk patients, scenarios where front-line testing is unrevealing, when conventional testing is unlikely to detect the suspected pathogen or suffers from long turnaround times (26).

The value of rapid cfmNGS tests is diminished if results are not interpreted effectively and acted upon promptly. Access and cost also remain major barriers to clinical implementation, limiting availability of NGS assays to well-resourced academic medical centers, and insurance coverage is inconsistent (27). Studies on universal, unchecked utilization of NGS show low diagnostic yield and lack of clinical utility (28–30). NGS tests are prone to overuse and misuse, which risks financial burden for patients and undermines confidence in such assays.

Diagnostic stewardship— thoughtful test selection grounded in a robust differential, ordered in the right clinical context, and interpreted through close collaboration between clinicians and microbiologists—is essential for maximizing the benefit of NGS assays. This case shows that NGS is only as useful as the clinical reasoning that guides its use.

## CONCLUSIONS

Since 2013, global outbreaks of invasive MC infections have been linked to contaminated HCDs used during cardiac surgery (6, 12, 31). MC infections can manifest years after exposure and carry mortality rates of 50%–70% (7, 15, 19). Despite warnings from the US Food and Drug Administration and recommended mitigation measures, the implicated devices (LivaNova) remain in use, and the infection control measures required to replace or decontaminate them may not be feasible in resource-limited settings (6). Clinicians should maintain a high index of suspicion for MC in patients with prior open-chest cardiac surgery who present with nonspecific systemic symptoms. Early suspicion guides appropriate testing, timely treatment, and improved outcomes.

Importantly, this case reminds us that traditional “old-school” microbiological testing- cultures, stains, and histopathology- remain indispensable. These widely accessible, cost-effective tools often yield clinically actionable results when guided by a rationale differential (Table 1). Continued efforts to refine and optimize these fundamental diagnostic tests remain essential, particularly for underserved communities that bear a disproportionate burden of infectious diseases.

## References

[1] Ladines-Lim JB, Yang W-T, Tebas P, O’Donnell J, Koenig H, Kreider E, Dyer K, Anwar M, Rodriguez E, Patel S, Rodino K, Glaser L, Richterman A. 2025. Delayed diagnosis of disseminated Mycobacterium intracellulare subsp. chimaera infective endocarditis via cell-free metagenomic next-generation sequencing: a case report. ASM Case Rep 1:e00003–25. doi:10.1128/asmcr.00003-25 41244979 PMC12530230

[2] Hasse B, Hannan MM, Keller PM, Maurer FP, Sommerstein R, Mertz D, Wagner D, Fernández-Hidalgo N, Nomura J, Manfrin V, et al.. 2020. International society of cardiovascular infectious diseases guidelines for the diagnosis, treatment and prevention of disseminated Mycobacterium chimaera infection following cardiac surgery with cardiopulmonary bypass. J Hosp Infect 104:214–235. doi:10.1016/j.jhin.2019.10.009 31715282

[3] Kasperbauer SH, Daley CL. 2019. Mycobacterium chimaera infections related to the heater–cooler unit outbreak: a guide to diagnosis and management. Clin Infect Dis 68:1244–1250. doi:10.1093/cid/ciy789 30371755

[4] Achermann Y, Rössle M, Hoffmann M, Deggim V, Kuster S, Zimmermann DR, Bloemberg G, Hombach M, Hasse B. 2013. Prosthetic valve endocarditis and bloodstream infection due to Mycobacterium chimaera. J Clin Microbiol 51:1769–1773. doi:10.1128/JCM.00435-13 23536407 PMC3716099

[5] Chandrasekar H, Hoganson DM, Lachenauer CS, Newburger JW, Sandora TJ, Saleeb SF. 2022. Mycobacterium chimaera outbreak management and outcomes at a large pediatric cardiac surgery center. Ann Thorac Surg 114:552–559. doi:10.1016/j.athoracsur.2021.07.074 34454904

[6] Tan NY, Tarabochia AD, DeSimone DC, DeSimone CV, Wilson JW, Bagameri G, Bennett CE, Abu Saleh OM. 2021. Updated experience of Mycobacterium chimaera infection: diagnosis and management in a tertiary care center. Open Forum Infect Dis 8:ofab348. doi:10.1093/ofid/ofab348 34377729 PMC8339283

[7] Julian KG, Crook T, Curley E, Appenheimer AB, Paules CI, Hasse B, Diekema DJ, Daley CL, de Sanctis J, Hellinger WC, Levin A, McSherry G, Freer C, Whitener CJ. 2020. Long-term follow-up of post-cardiac surgery Mycobacterium chimaera infections: a 5-center case series. J Infect 80:197–203. doi:10.1016/j.jinf.2019.12.007 31863789

[8] Mason M, Gregory E, Foster K, Klatt M, Zoubek S, Eid AJ. 2022. Pharmacologic management of Mycobacterium chimaera infections: a primer for clinicians. Open Forum Infect Dis 9:ofac287. doi:10.1093/ofid/ofac287 35866101 PMC9297092

[9] Zweifel SA, Mihic-Probst D, Curcio CA, Barthelmes D, Thielken A, Keller PM, Hasse B, Böni C. 2017. Clinical and histopathologic ocular findings in disseminated Mycobacterium chimaera infection after cardiothoracic surgery. Ophthalmology 124:178–188. doi:10.1016/j.ophtha.2016.09.032 27871762

[10] Sax H, Bloemberg G, Hasse B, Sommerstein R, Kohler P, Achermann Y, Rössle M, Falk V, Kuster SP, Böttger EC, Weber R. 2015. Prolonged outbreak of Mycobacterium chimaera infection after open-chest heart surgery. Clin Infect Dis 61:67–75. doi:10.1093/cid/civ198 25761866

[11] Kohler P, Kuster SP, Bloemberg G, Schulthess B, Frank M, Tanner FC, Rössle M, Böni C, Falk V, Wilhelm MJ, et al.. 2015. Healthcare-associated prosthetic heart valve, aortic vascular graft, and disseminated Mycobacterium chimaera infections subsequent to open heart surgery. Eur Heart J 36:2745–2753. doi:10.1093/eurheartj/ehv342 26188001

[12] Ben Appenheimer A, Diekema DJ, Berriel-Cass D, Crook T, Daley CL, Dobbie D, Edmond M, Hellinger W, Ince D, Julian KG, Lampen R, Arbulu R, Cooper E, Curley E III, De Sanctis J, Freer C, Strong M, Gajurel K, Hasan N, Walker S, Whitener C. 2016. Mycobacterium chimaera outbreak response: experience from four United States healthcare systems. Open Forum Infect Dis 3:2392. doi:10.1093/ofid/ofw195.10

[13] Wilson WR, Bower TC, Creager MA, Amin-Hanjani S, O’Gara PT, Lockhart PB, Darouiche RO, Ramlawi B, Derdeyn CP, Bolger AF, Levison ME, Taubert KA, Baltimore RS, Baddour LM, American Heart Association Committee on Rheumatic Fever, Endocarditis, and Kawasaki Disease of the Council on Cardiovascular Disease in the Young; Council on Cardiovascular and Stroke Nursing; Council on Cardiovascular Radiology and Intervention; Council on Cardiovascular Surgery and Anesthesia; Council on Peripheral Vascular Disease; and Stroke Council. 2016. Vascular graft infections, mycotic aneurysms, and endovascular infections: a scientific statement from the American heart association. Circulation 134:e412–e460. doi:10.1161/CIR.0000000000000457 27737955

[14] Trauth J, Matt U, Kohl TA, Niemann S, Herold S. 2023. Blind spot in endocarditis guidelines: Mycobacterium chimaera prosthetic valve endocarditis after cardiac surgery-a case series. Eur Heart J Case Rep 7:ytad400. doi:10.1093/ehjcr/ytad400 37654802 PMC10468014

[15] Scriven JE, Scobie A, Verlander NQ, Houston A, Collyns T, Cajic V, Kon OM, Mitchell T, Rahama O, Robinson A, et al.. 2018. Mycobacterium chimaera infection following cardiac surgery in the United Kingdom: clinical features and outcome of the first 30 cases. Clin Microbiol Infect 24:1164–1170. doi:10.1016/j.cmi.2018.04.027 29803845

[16] Ganatra S, Sharma A, D’Agostino R, Gage T, Kinnunen P. 2018. Mycobacterium chimaera mimicking sarcoidosis. Methodist Debakey Cardiovasc J 14:301–302. doi:10.14797/mdcj-14-4-301 30788017 PMC6369614

[17] Tan N, Sampath R, Abu Saleh OM, Tweet MS, Jevremovic D, Alniemi S, Wengenack NL, Sampathkumar P, Badley AD. 2016. Disseminated Mycobacterium chimaera infection after cardiothoracic surgery. Open Forum Infect Dis 3:ofw131. doi:10.1093/ofid/ofw131 27703994 PMC5047393

[18] Trautman C, Da Costa JR, Cortese C, Aslam N. 2020. Prosthetic valve endocarditis from Mycobacterium chimaera infection causing granulomatous interstitial nephritis. IDCases 20:e00733. doi:10.1016/j.idcr.2020.e00733 32154105 PMC7057185

[19] Park Y, Lee EH, Jung I, Park G, Kang YA. 2019. Clinical characteristics and treatment outcomes of patients with macrolide-resistant Mycobacterium avium complex pulmonary disease: a systematic review and meta-analysis. Respir Res 20:286. doi:10.1186/s12931-019-1258-9 31852452 PMC6921583

[20] Diel R, Nienhaus A, Ringshausen FC, Richter E, Welte T, Rabe KF, Loddenkemper R. 2018. Microbiologic outcome of interventions against Mycobacterium avium complex pulmonary disease: a systematic review. Chest 153:888–921. doi:10.1016/j.chest.2018.01.024 29410162

[21] Kobashi Y, Abe M, Mouri K, Obase Y, Kato S, Oka M. 2012. Relationship between clinical efficacy for pulmonary MAC and drug-sensitivity test for isolated MAC in a recent 6-year period. J Infect Chemother 18:436–443. doi:10.1007/s10156-011-0351-x 22205543

[22] Kobashi Y, Yoshida K, Miyashita N, Niki Y, Oka M. 2006. Relationship between clinical efficacy of treatment of pulmonary Mycobacterium avium complex disease and drug-sensitivity testing of Mycobacterium avium complex isolates. J Infect Chemother 12:195–202. doi:10.1007/s10156-006-0457-8 16944258

[23] Lau D, Cooper R, Chen J, Sim VL, McCombe JA, Tyrrell GJ, Bhargava R, Adam B, Chapman E, Croxen MA, Garady C, Antonation K, van Landeghem FKH, Ip S, Saxinger L. 2020. Mycobacterium chimaera encephalitis following cardiac surgery: a new syndrome. Clin Infect Dis 70:692–695. doi:10.1093/cid/ciz497 31247065

[24] Xu Z. 2016. Disseminated MAC infection with marrow noncaseating granuloma. Blood 128:2476. doi:10.1182/blood-2016-08-732230 27856473

[25] Lecorche E, Haenn S, Mougari F, Kumanski S, Veziris N, Benmansour H, Raskine L, Moulin L, Cambau E, Aubry A, Brossier F, Chauffour A, Jaffre J, Jarlier V, Robert J, Sougakoff W. 2018. Comparison of methods available for identification of Mycobacterium chimaera. Clin Microbiol Infect 24:409–413. doi:10.1016/j.cmi.2017.07.031 28782649

[26] Gaston DC, Chiang AD, Dee K, Dulek D, Banerjee R, Humphries RM. 2024. Diagnostic stewardship for next-generation sequencing assays in clinical microbiology: an appeal for thoughtful collaboration. Clin Lab Med 44:63–73. doi:10.1016/j.cll.2023.10.002 38280798

[27] Shean RC, Garrett E, Malleis J, Lieberman JA, Bradley BT. 2024. A retrospective observational study of mNGS test utilization to examine the role of diagnostic stewardship at two academic medical centers. J Clin Microbiol 62:e00605–24. doi:10.1128/jcm.00605-24 39162437 PMC11389146

[28] Shishido AA, Noe M, Saharia K, Luethy P. 2022. Clinical impact of a metagenomic microbial plasma cell-free DNA next-generation sequencing assay on treatment decisions: a single-center retrospective study. BMC Infect Dis 22:372. doi:10.1186/s12879-022-07357-8 35418022 PMC9006594

[29] Hogan CA, Yang S, Garner OB, Green DA, Gomez CA, Dien Bard J, Pinsky BA, Banaei N. 2021. Clinical impact of metagenomic next-generation sequencing of plasma cell-free DNA for the diagnosis of infectious diseases: a multicenter retrospective cohort study. Clin Infect Dis 72:239–245. doi:10.1093/cid/ciaa035 31942944

[30] Weiss ZF, Pyden AD, Jhaveri TA, Kanjilal S. 2023. The diagnostic and clinical utility of microbial cell-free DNA sequencing in a real-world setting. Diagn Microbiol Infect Dis 107:116004. doi:10.1016/j.diagmicrobio.2023.116004 37467522

[31] Lyman MM, Grigg C, Kinsey CB, Keckler MS, Moulton-Meissner H, Cooper E, Soe MM, Noble-Wang J, Longenberger A, Walker SR, Miller JR, Perz JF, Perkins KM. 2017. Invasive nontuberculous mycobacterial infections among cardiothoracic surgical patients exposed to heater–cooler devices^1^. Emerg Infect Dis 23:796–805. doi:10.3201/eid2305.161899 28418290 PMC5403026

